# Phytochemical Analysis of *Pfaffia glomerata* Inflorescences by LC-ESI-MS/MS

**DOI:** 10.3390/molecules191015720

**Published:** 2014-09-29

**Authors:** Daniele F. Felipe, Lara Z. S. Brambilla, Carla Porto, Eduardo J. Pilau, Diógenes A. G. Cortez

**Affiliations:** 1 Pharmaceutical Sciences Postgraduate Program, Department of Pharmacy, State University of Maringá, Av. Colombo, 5790, Maringá, Paraná 87020-900, Brazil; E-Mails: daniele.felipe@uol.com.br (D.F.F.); larazampar@yahoo.com.br (L.Z.S.B.); 2 Department of Chemistry, State University of Maringá, Av. Colombo, 5790, Maringá, Paraná 87020-900, Brazil; E-Mails: cporto.silva@gmail.com (C.P.); ejpilau@uem.br (E.J.P.)

**Keywords:** *Pfaffia glomerata*, inflorescences, LC-ESI-MS/MS, β-ecdysone, flavonoid glycosides, triterpenoid saponins

## Abstract

*Pfaffia glomerata* contains high levels of β-ecdysone, which has shown a range of beneficial pharmacological effects. The present study demonstrated that inflorescences of *P. glomerata* contain other important bioactive compounds in addition to β-ecdysone. The identification of compounds from inflorescences using liquid chromatography coupled with electrospray ionization tandem mass spectrometry (LC-ESI-MS/MS) was performed for the first time. The eight compounds identified were β-ecdysone, flavonoid glycosides such as quercetin-3-*O*-glucoside, kaempferol-3-*O*-glucoside and kaempferol-3-*O*-(6-*p*-coumaroyl)-glucoside, oleanane-type triterpenoid saponins such as ginsenoside Ro and chikusetsusaponin IV, in addition to oleanonic acid and gluconic acid. This study provided information on the phytochemicals contained in *P. glomerata* inflorescences revealing the potential application of this plant part as raw material for the phytotherapeutic and cosmetic industries.

## 1. Introduction

*Pfaffia glomerata* (Spreng.) Pedersen (Amaranthaceae) is a perennial herb, traditionally known as Brazilian ginseng and its roots are widely used in Brazilian traditional medicine [1,2]. Several pharmaceutical manufacturers have produced phytopharmaceuticals containing *P. glomerata*, although only the roots have been processed to obtain the active ingredients [3,4]. The extracts show several biological properties including, gastroprotective effects [5], antioxidant activity [6,7], leishmanicidal potential [8], anti-inflammatory activity, and analgesic effect [9].

Several important compounds have been isolated and identified from roots of *P. glomerata*, such as glomeric acid (a triterpenoid) and pfameric acid (a nortriterpenoid), together with ecdysterone (β-ecdysone), rubrosterone, oleanolic acid, and β-glucopyranosyl oleanolate [10]. β-Ecdysone is the most important phytoecdysteroid in *P. glomerata* [11]. The β-ecdysone content in *P. glomerata* has been analyzed almost exclusively in roots, which contain large amounts of this compound [5,7,12]. However, recent studies have confirmed the presence of significant amounts β-ecdysone in all of the major parts of *P. glomerata*, including stems and inflorescences [3,13]. Many pharmacological effects are attributed to β-ecdysone, such as antioxidant activity, and wound-healing and skin-regenerating properties, in addition to its use in cosmetic preparations [6,14,15,16].

Although the phytochemical content of the roots of *P. glomerata* has been extensively investigated, information about the chemical compounds of other parts of this plant is still sparse. In this study, we analyzed *P. glomerata* inflorescences using the LC-ESI-MS/MS method, which led to the identification of important bioactive compounds. To our knowledge, this is the first report on the identification of compounds in *P. glomerata* inflorescences by LC-ESI-MS/MS analysis. This study demonstrates the potential value of these inflorescences for cosmetic and medicinal applications.

## 2. Results and Discussion

LC-ESI-MS/MS analyses were performed in positive and negative ionization modes to obtain maximum information on the composition of the inflorescences of *P. glomerata*. β-ecdysone was used as a standard because it is the main compound reported in *P. glomerata*.

**Figure 1:**
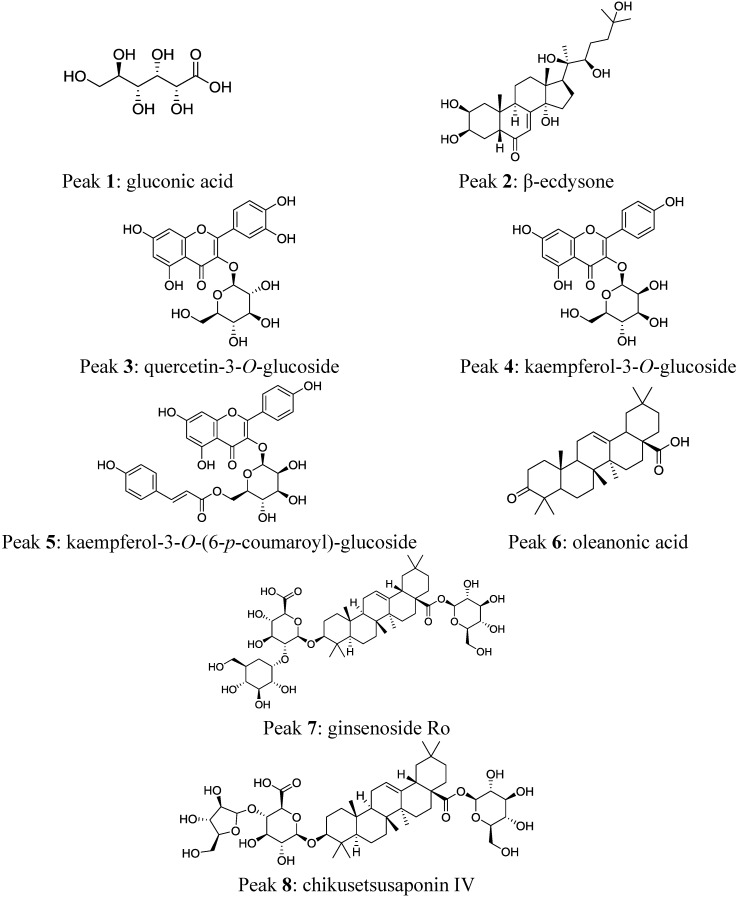
Chemical structures of the eight compounds proposed in *P. glomerata* inflorescences by LC-ESI-MS/MS.

Compounds were characterized based on their mass spectra, using the precursor ion, fragment ions, and comparison of the fragmentation patterns with molecules described in the literature. Their chemical structures are shown in Figure 1, and the putative identification these compounds is discussed below and summarized in Table 1, where the compounds are numbered according to their retention times in the total ion chromatograms (TICs).

### 2.1. Identification of Compounds in P. glomerata Inflorescences by LC-ESI-MS/MS in Positive Ion Mode

**Figure 2:**
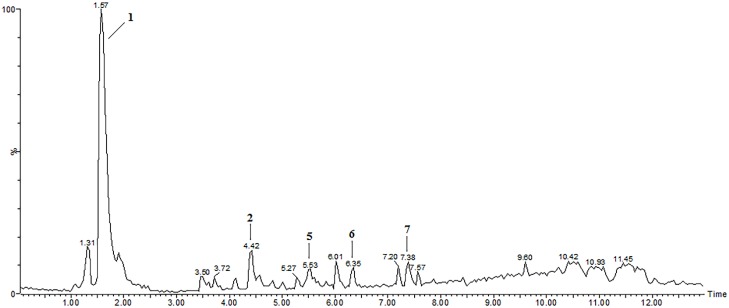
LC-ESI-MS total ion chromatogram (TIC) of the extract of *Pfaffia glomerata* inflorescences showing peaks **1**, **2**, **5**, **6** and **7** detected in the positive ion mode.

Figure 2 shows the total ion chromatogram (TIC) of the extract of *P. glomerata* inflorescences acquired in the positive ion mode. Peak **1** with a retention time (t_r_) of 1.6 min (Table 1 and Figure 2) was proposed as gluconic acid that produced an [M+K]^+^ ion at *m/z* 235 by forming an adduct with a potassium ion. In addition, fragment ions at *m/z* 118 and at *m/z* 59 were observed in the ESI-MS/MS spectrum.

**Table 1:** Proposed compounds detected in *Pfaffia glomerata* inflorescences by LC-ESI-MS/MS in positive and negative ion modes.

Peak ^a^	t_r_ (min)	Positive Ion Mode		Negative Ion Mode		Tentative Assignment
		Precursor Ion (*m/z*)	Fragment Ions ^d^ (*m/z*)	Precursor Ion (*m/z*)	Fragment Ions ^d^ (*m/z*)	
1	1.6	235 [M+K]^+ c^	**118**, 59	195 [M-H]^−^	177, 159, 129, 99, **75**	gluconic acid
2	4.4	481 [M+H]^+ b^	463, 445, 427, 409, **371**, 165	525 [M+HCOO]^− c^	**479**, 461, 319, 159	β-ecdysone
3	4.7	ND	ND	463 [M-H]^−^	**301/300**	quercetin-3-*O*-glucoside
4	4.9	ND	ND	447 [M-H]^−^	**284**	kaempferol-3-*O*-glucoside
5	5.5	595 [M+H]^+^	309, 287, **147**	593 [M-H]^−^	447, 307, **285**	kaempferol-3-*O*-(6-*p*-coumaroyl)-glucoside
6	6.4	455 [M+H]^+^	438, 410, **147**	ND	ND	oleanonic acid
7	7.4	439 [aglycone+H-H_2_O]^+^	393, 249, **203**, 191	955 [M-H]^−^	910, **793**, 631	ginsenoside Ro
8	8.6	ND	ND	925 [M-H]^−^	**793**,763	chikusetsusaponin IV

^a^ Peak number, refer to Figure 2 and Figure 4; ^b^ Compared with reference standard; ^c^ Adduct ions; ^d^ The base peaks in MS/MS spectra are in bold; ND: not detected.

Kim *et al*. [17] showed that gluconic acid forms sodium adducts. In the positive mode, acids commonly form adducts with the cations in the sample or mobile phase, and in the negative ionization mode, they deprotonate easily [18]. Gluconic acid is produced from glucose, and occurs naturally in fruit, honey and wine [19,20]. Gluconic acid and its derivatives have wide applications in the food and pharmaceutical industries [19].

**Figure 3:**
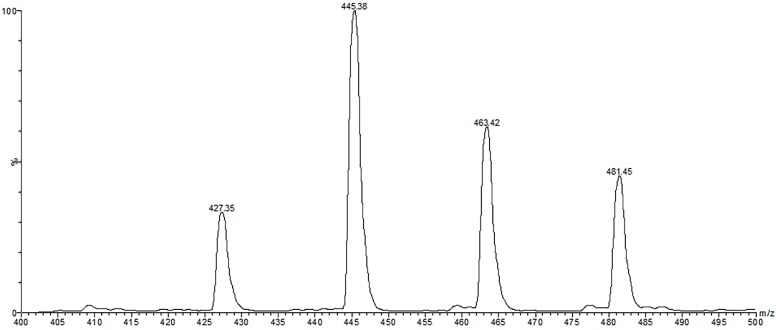
Direct-infusion ESI-MS/MS spectrum of β-ecdysone standard showing [M+H]^+^, [M+H-H_2_0]^+^, [M+H-2H_2_0]^+^, and [M+H-3H_2_0]^+^ in positive ion mode.

Peak **2** with t_r_ of 4.4 min (Table 1 and Figure 2) was identified as β-ecdysone with [M+H]^+^ ion at *m/z* 481 and fragment ions at *m/z* 463, 445, 427 and 409, which correspond to the successive loss of 1–4 molecules of water. This is in agreement with the ESI-MS/MS spectrum obtained for the β-ecdysone standard (Figure 3). These fragment ions associated with water loss were also observed in the MS/MS spectrum of β-ecdysone in previous studies [21,22,23,24]. Wainwright *et al*. [25] reported that for free ecdysteroids, the major ions observed in the spectra are the protonated pseudomolecular ion [M+H]^+^ and ions corresponding to the successive losses of water molecules, [M+H-*n*H_2_0]^+^ (*n* = 1–4).

This loss of water could arise from the OH group at position C-25 and C-14, which could be explained by a proton-bridging effect on the 20,22-diol function and 2,3-diol function, respectively [22,23,26]. In addition, the protonation of C25-OH followed by loss of water is favored due to the formation of a stable tertiary carbonium ion, which is also produced from the C14-OH [22,24]. Other characteristic fragment ions at *m/z* 371 and at *m/z* 165 were observed in the ESI-MS/MS spectrum obtained for peak **2**, as also reported in previous studies [23,26,27]. The fragment ion with *m/z* 371 was produced from cleavage between C-23 and C-24 in the side chain, followed by the loss of two molecules of water, as described by Destrez *et al*. [26].

Peak **5** with t_r_ of 5.5 min (Table 1 and Figure 2) was proposed as kaempferol-3-*O*-(6-*p*-coumaroyl)-glucoside (tiliroside) based on the [M+H]^+^ ion at *m*/*z* 595 and fragment ions at *m/z* 287 and at *m/z* 309, which are characteristic of kaempferol and the coumaroylglucoside moiety, respectively [28,29,30,31]. Another fragment ion was observed at *m/z* 147 [coumaroyl+H]^+^, which is typical of the presence of coumaroyl moieties, according to Karioti *et al*. [32]. Kaempferol-3-*O*-(6-*p*-coumaroyl)-glucoside is formed by acylation of the sugar moiety with hydroxycinnamic acid [31,32,33]. Previous studies showed that acylated flavonoid glycosides have higher antioxidant and antibacterial activities than their corresponding glycosides [34].

Peak **6** with t_r_ of 6.4 min (Table 1 and Figure 2), which produced an [M+H]^+^ ion at *m/z* 455 in the full MS scan, was tentatively identified as oleanonic acid. The ESI-MS/MS spectrum showed characteristic fragment ions such as [M+H-OH]^+^ ion at *m/z* 438 due to the loss of a hydroxyl group, and a fragment ion at *m/z* 410 [M+H-CO_2_H]^+^ corresponding to the loss of CO_2_H, as previously described, in addition to a fragment ion at *m/z* 147 [35].

Peak **7** with t_r_ of 7.4 min (Table 1 and Figure 2) was suggested as oleanolic acid aglycone based on the ESI-MS/MS spectrum, which showed a fragment ion at *m/z* 439 [M+H-H_2_O]^+^ corresponding to the loss of one molecule of water, a fragment ion at *m/z* 393 [M+H-H_2_O-HCO_2_H]^+^ due to the loss of formic acid, in addition to fragment ions at *m/z* 249 [C_16_H_25_O_2_]^+^, at *m/z* 203 [C_15_H_23_]^+^ and at *m/z* 191 [C_14_H_23_]^+^, which is in agreement with the literature [35,36,37,38]. Oleanolic acid and its derivatives were identified in roots of *P. glomerata* by Shiobara *et al*. [10]. The oleanane-type triterpenoid saponins have oleanolic acid as an aglycone skeleton; product ions of [M+H-H_2_O]^+^ at *m/z* 439 formed by retro-Diels-Alder (RDA) fragmentation in positive ion mode are useful to screen these saponins, as described by Li *et al*. [38].

### 2.2. Identification of Compounds in P. glomerata Inflorescences by LC-ESI-MS/MS in Negative Ion Mode

**Figure 4:**
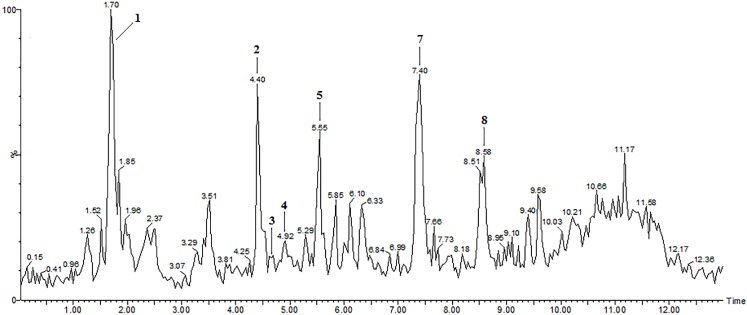
LC-ESI-MS total ion chromatogram (TIC) of the extract of *Pfaffia glomerata* inflorescences showing peaks **1**, **2**, **3**, **4**, **5**, **7** and **8** detected in the negative ion mode.

In the LC-ESI-MS/MS analysis of the extract of *P. glomerata* inflorescences using the negative ion mode, peak **1**, which was proposed as gluconic acid in the positive mode (Table 1 and Figure 4), demonstrated an [M-H]^−^ ion at *m*/*z* 195 and fragment ions at *m/z* 177, *m/z* 159, *m/z* 129, *m/z* 99 and *m/z* 75 in the ESI-MS/MS spectrum. These are characteristic fragment ions of this compound [39,40,41].

Peak **2** with t_r_ of 4.4 min (Table 1 and Figure 4), which as confirmed as β-ecdysone in the positive mode, exhibited the adduct ion [M+HCOO]^−^ at *m/z* 525 in the negative mode, due to the addition of 0.1% formic acid in the mobile phase, in agreement with previous studies [23,42]. The ESI-MS/MS spectrum showed the molecular ion [M-H]^−^ at *m/z* 479, and a fragment ion at *m/z* 461 due to the loss of one molecule of water, as described by Stevens *et al*. [23] and Destrez *et al*. [43]. Other fragment ions were observed at *m/z* 319 and *m/z* 159 resulting from cleavage on the side chain between C17 and C20 in β-ecdysone, as previously reported [23,26,43].

In addition, the LC-ESI-MS/MS analysis of the extract of *P. glomerata* in the negative ion mode showed three flavonol derivatives. Peak **3** with t_r_ of 4.7 min (Table 1 and Figure 4) was tentatively assigned to quercetin-3-*O*-glucoside (isoquercitrin) with an [M-H]^–^ ion at *m/z* 463 and a fragment ion [quercetin-H]^–^ at *m/z* 301 obtained after loss of a glucose unit (−162 amu). This is in agreement with the ESI-MS/MS spectrum shown in previous studies on other plant species [44,45,46,47,48,49].

The ESI-MS/MS spectrum of quercetin-3-*O*-glucoside also showed a fragment ion at *m/z* 300, which is characteristic of the deprotonated radical aglycone ion [quercetin-H]^−^, formed by the homolytic cleavage of the *O*-glycosidic bond, and has been proposed as indicative of quercetin glycosides [46,50]. Hvattum and Ekeberg [46] reported that flavonoid glycosides can undergo collision-induced homolytic and heterolytic cleavages of the *O*-glycosidic bond, producing deprotonated radical aglycone ((Y_0_-H)^−^) and aglycone (Y_0_^−^) product ions, depending on the structure of the flavonoid glycosides, and the nature and the position of the sugar substitution. In the case of quercetin-3-*O*-glucoside, the aglycone fragment appeared at *m*/*z* 301 and the radical aglycone ion at *m*/*z* 300.

Two peaks were tentatively assigned to kaempferol derivatives based on their ESI-MS/MS spectra producing the kaempferol aglycone at *m/z* 285 in negative mode. Peak **4** with t_r_ of 4.9 min (Table 1 and Figure 4) with an [M-H]^–^ ion at *m/z* 447, and a fragment ion [kaempferol-2H]^–^ at *m/z* 284 resulting from the loss of a glucose moiety (−162 amu), was suggested to be kaempferol-3-*O*-glucoside (astragalin). Our results are completely in agreement with those reported in previous studies [31,45,48,49,51].

Sánchez-Rabaneda *et al*. [44] demonstrated that the ESI-MS/MS spectra for flavonol *O*-glycosides such as hyperoside, isoquercitrin (quercetin-3-*O*-glucoside), quercitrin and kaempferol-3-*O*-glucoside show the deprotonated molecule [M-H]^–^ of the glycoside and the ion corresponding to the deprotonated aglycone [A-H]^–^, which is formed by loss of the rhamnose, glucose or galactose moiety from the glycosides. A previous study of the MS fragmentation of flavonol-3-*O*-glycosides demonstrated that the presence in the MS^2^ and/or MS^3^ of the deprotonated ion from their aglycones, at *m/z* 284/285 and 300/301, indicates that the compounds are kaempferol-3-*O*- and quercetin-3-*O*- derivatives, respectively [52]. In our study, the compounds proposed as quercetin-3-*O*-glucoside and kaempferol-3-*O*-glucoside were detected only in negative mode due to the acidic nature of these compounds, which made the ions more abundant and easily detected in this ionization mode, in agreement with the literature [53]. According to Gouveia and Castilho [54], the use of ESI operating in the negative mode has proven to be more selective and efficient in the characterization of phenolic compounds, even those present in trace amounts.

Peak **5** with t_r_ of 5.5 min (Table 1 and Figure 4) showed an [M-H]^−^ ion at *m*/*z* 593 and a fragment ion at *m/z* 285 [kaempferol-H]^−^ due to the loss of a coumaroylglucoside moiety (−308 amu), in addition to fragment ions at *m/z* 447 [M-*p*-coumaroyl]^−^ and at *m/z* 307 [M-kaempferol]^−^ suggesting the compound kaempferol-3-*O*-(6-*p*-coumaroyl)-glucoside, which was also tentatively identified in the positive ion mode [30,31,32].

As far as we are aware, no reports about flavonoid glycosides in *P. glomerata* inflorescences have previously been published. Although these three flavonoid glycosides have been proposed, their isomers could also be suggested too. According to Plazonić *et al*. [55], flavonoid glycosides have many isomers with the same molecular weight but different aglycone and sugar components at different positions on the aglycone ring. Kite and Veitch [56], reported that determining the identity of the sugars, and the manner in which they are linked, by mass spectrometry alone is challenging. For example, the loss of 162 amu indicates a hexose sugar but does not specify whether that sugar is glucose or galactose.

Peak **7** with t_r_ of 7.4 min (Table 1 and Figure 4), which was suggested in the positive mode to be the aglycone oleanolic acid, might be tentatively assigned to an oleanane-type triterpenoid saponin such as ginsenoside Ro (3-*O*-β-d-glucopyranosyl-(1→2)-β-d-glucuronopyranosyl-28-*O*-β-d-glucopyranosyl oleanolic acid) or its isomer. The ESI-MS/MS spectrum yielded the [M-H]^−^ ion at *m/z* 955, a fragment ion at *m/z* 910 [M-H-CO_2_H]^−^ due to loss of formic acid, and characteristic fragment ions at *m/z* 793 [M-H-glucose]^−^ and at *m/z* 631 [M-H-2glucose]^−^ corresponding to loss of a glucose unit (−162 amu) at C-28 and to subsequent loss of another terminal glucose moiety at C-3, respectively, as reported in the literature [38,57,58]. Most fragments of triterpenoid saponins are derived from the sugar moieties in the negative mode, while the fragments originate mostly from the sapogenins (aglycones) in the positive mode [59]. The sugar moiety of oleanane-type triterpenoid saponins is important for their bioactivities [60].

The ESI-MS/MS spectrum (of the compound corresponding to peak **8** with t_r_ of 8.6 min (Table 1 and Figure 4), showed an [M-H]^−^ ion at *m/z* 925 and fragment ions at *m/z* 793 [M-H-132]^−^ and at *m/z* 763 [M-H-162]^−^ attributed to loss of an arabinose moiety and a glucose moiety, respectively. These results suggest that the compound might be an oleanane-type triterpenoid saponin such as chikusetsusaponin IV (3-*O*-α-L-arabinopyranosyl-(1→4)-β-d-glucuronopyranosyl-28-*O*-β-d-gluco-pyranosyl oleanolic acid) or its isomers, according to the literature [38,61]. Further investigations by NMR methods are needed to confirm the absolute configuration of the isomeric compounds, especially to define the position of the sugar moiety.

Therefore in the present study, the main compounds proposed in *P. glomerata* inflorescences were β-ecdysone and flavonoid glycosides, in addition to gluconic acid and triterpenoid derivatives such as saponins and oleanonic acid. If *P. glomerata* inflorescences are a source of important bioactive compounds, their utilization in phytotherapeutics and in the cosmetic industry should be encouraged.

## 3. Experimental Section

### 3.1. Chemicals and Materials

The methanol used for sample preparation was purchased from Merck (analytical grade; Darmstadt, Germany). HPLC grade acetonitrile (Merck), ultrapure water (Milli-Q system; Millipore, Bedford, MA, USA) and formic acid (Merck) were used for mobile phase preparation in the LC-ESI-MS/MS analysis. β-ecdysone (Chromadex, Irvine, CA, USA) was used as a standard.

### 3.2. Plant Material and Sample Preparation

*Pfaffia glomerata* was collected in April 2010 in Querência do Norte (23°05'02''S; 53°29'02''W), Paraná, Brazil. The plant was identified by Dr. Maria Salete Marchioretto, and a voucher specimen (PACA 107100) was deposited at the Herbarium PACA, Universidade do Vale do Rio dos Sinos, Rio Grande do Sul, Brazil. The plant was dried in a circulating-air oven at 45 °C. The inflorescences of the plant were then separated, triturated in a knife mill, and stored.

The extract obtained from the inflorescences was prepared using the Soxhlet method, with 10 g of plant material and a solution of ethanol:water (9:1 v/v) as established by Serra *et al*. [13]. The extract was filtered, and the organic solvent was removed under vacuum at 40 °C, using a rotary evaporator, and lyophilized.

The *P. glomerata* extract and β-ecdysone standard were dissolved in methanol at concentrations of 3000 µg/mL and 500 µg/mL, respectively. The solutions were filtered through a 0.45 mm membrane filter (Millipore, Bedford, MA, USA).

### 3.3. LC-ESI-MS and LC-ESI-MS/MS Analyses

A Waters 2489 HPLC system coupled to a Micromass Quattro micro API mass spectrometer (Waters, Milford, MA, USA), triple quadrupole mass analyzer, with an electrospray ionization source (ESI) was used to perform the ESI-MS and ESI-MS/MS analyses, which were controlled using MassLynx 4.1 software (Waters, Milford, MA, USA).

The chromatographic separations were performed using a Symmetry C18 column (i.d., 3.5 µm; 75 × 4.6 mm) maintained at room temperature. The mobile phase consisted of 0.1% formic acid in water (solvent A) and 0.1% formic acid in acetonitrile (solvent B). The gradient system was as follows: 0 min 2% solvent B; 9 min 98% solvent B, kept for 1 min; 12 min 2% solvent B, remaining in this last condition for 1 min. The flow rate was 0.5 mL/min and the sample injection volume was 10 µL.

The mass spectrometer operating conditions were: capillary voltage +2.5 kV (positive ionization mode) and −2.5 kV (negative ionization mode); cone voltage from 20 to 35 V. The source temperature and desolvation gas temperature were set at 150 and 450 °C, respectively. The cone gas flow and desolvation gas flow were 25 and 900 L/h, respectively. Spectra were recorded in positive and negative ion modes. Full-scan spectra were acquired over the range of *m/z* 100–1000. In the second MS, the most intense ions from the first MS spectrum were selected for the collision-induced dissociation (MS experiment). The standard solution of β-ecdysone was directly infused (10 µL/min) into the ESI interface using the positive ion mode. The MS information was interpreted and compared with spectra available in the literature.

## 4. Conclusions

This is the first study to examine the chemical composition of *P. glomerata* inflorescences, determined by liquid chromatography coupled with electrospray ionization tandem mass spectrometry (LC-ESI-MS/MS). The eight compounds proposed were β-ecdysone (peak **2**), flavonoid glycosides such as quercetin-3-*O*-glucoside (peak **3**), kaempferol-3-*O*-glucoside (peak **4**) and kaempferol-3-*O*-(6-*p*-coumaroyl)-glucoside (peak **5**), oleanane-type triterpenoid saponins such as ginsenoside Ro (peak **7**) and chikusetsusaponin IV (peak **8**), in addition to oleanonic acid (peak **6**) and gluconic acid (peak **1**). To our knowledge, this is the first report of these compounds in *P. glomerata* inflorescences, except for β-ecdysone. This study contributes to promote the use of this plant part in phytotherapeutics and in the cosmetic industry, due to the important biologically active compounds that were identified. The inflorescences could be used for extraction of these bioactive compounds, rather than being discarded during the processing of *P. glomerata* roots.

## References

[1] Nascimento E.X., Mota J.H., Vieira M.C., Zárate N.A.H. (2007). Produção de biomassa de *Pfaffia glomerata* (Spreng.) Pedersen e *Plantago major* L. em cultivo solteiro e consorciado. Cienc. Agrotec..

[2] Festucci-Buselli R.A., Contim L.A.S., Barbosa L.C.A., Stuart J.J., Otoni W.C. (2008). Biosynthesis and potencial functions of the ecdysteroid 20-hydroxyecdysone—A review. Botany.

[3] Flores R., Brondani D., Cezarotto V., Giacomelli S.R., Nicoloso F.T. (2010). Micropropagation and β-ecdysone content of the Brazilian ginsengs *Pfaffia glomerata* and *Pfaffia tuberosa*. In Vitro Cell. Dev. Biol.-Plant.

[4] Carulo M.F. (2012). Use of SFC in extraction of adaptogens from Brazilian plants. Am. J. Anal. Chem..

[5] Freitas C.S., Baggio C.H., da Silva-Santos J.E., Rieck L., Santos C.A.M., Junior C.C., Ming L.C., Cortez D.A.G., Marques M.C.A. (2004). Involvement of nitric oxide in the gastroprotective effects of an aqueous extract of *Pfaffia glomerata* (Spreng) Pedersen, Amaranthaceae, in rats. Life Sci..

[6] Daniel J.F.S., Alves K.Z., Jacques J.D.S., Souza P.V.S., Carvalho M.G., Freire R.B., Ferreira D.T., Freire M.F.I. (2005). Free radical scavenging activity of *Pfaffia glomerata* (Spreng.) Pedersen (Amaranthaceae). Indian J. Pharmacol..

[7] Leal P.F., Kfouri M.B., Alexandre F.C., Fagundes F.H.R., Prado J.M., Toyama M.H., Meireles M.A.A. (2010). Brazilian Ginseng extraction via LPSE and SFE: Global yields, extraction kinetics, chemical composition and antioxidant activity. J. Supercrit. Fluids.

[8] Neto A.G., da Silva Filho A.A., Costa J.M.L.C., Vinholis A.H.C., Souza G.H.B., Cunha W.R., Silva M.L.A., Albuquerque S., Bastos J.K. (2004). Evaluation of the trypanocidal and antileishmanial *in vitro* activity of the crude hydroalcoholic extract of *Pfaffia glomerata* (Amaranthaceae) roots. Phytomedicine.

[9] Neto A.G., Costa J.M., Belati C.C., Vinholis A.H., Possebom L.S., da Silva Filho A.A., Cunha W.R., Carvalho J.C., Bastos J.K., Silva M.L. (2005). Analgesic and anti-inflammatory activity of a crude root extract of *Pfaffia glomerata* (Spreng) Pedersen. J. Ethnopharmacol..

[10] Shiobara Y., Inoue S.S., Kato K., Nishiguchi Y., Oishi Y., Nishimoto N., de Oliveira F., Akisue G., Akisue M.K., Hashimoto G. (1993). A nortriterpenoid, triterpenoids and ecdystereoids from *Pfaffia glomerata*. Phytochemistry.

[11] Iarema L., da Cruz A.C.F., Saldanha C.W., Dias L.L.C., Vieira R.F., de Oliveira E.J., Otoni W.C. (2012). Photoautotrophic propagation of Brazilian ginseng [*Pfaffia glomerata* (Spreng.) Pedersen]. Plant Cell Tissue Org..

[12] Zimmer A.R., Bruxel F., Bassani V.L., Gosmann G. (2006). HPLC method for the determination of ecdysterone in extractive solution from *Pfaffia glomerata*. J. Pharm. Biomed..

[13] Serra L.Z., Felipe D.F., Cortez D.A.G. (2012). Quantification of β-ecdysone in differents parts of *Pfaffia glomerata* by HPLC. Rev. Bras. Farmacogn..

[14] Lafont R., Dinan L. (2003). Practical uses for ecdysteroids in mammals including humans: An update. J. Insect Sci..

[15] Nsimba R.Y., Kikuzaki H., Konishi Y. (2008). Ecdysteroids act as inhibitors of calf skin collagenase and oxidative stress. J. Biochem. Mol. Toxicol..

[16] Dinan L., Lafont R. (2006). Effects and applications of arthropod steroid hormones (ecdysteroids) in mammals. J. Endocrinol..

[17] Kim H.Y., Park H.M., Lee C.H. (2012). Mass spectrometry-based chemotaxonomic classification of *Penicillium* species (*P. echinulatum*, *P. expansum*, *P. solitum*, and *P. oxalicum*) and its correlation with antioxidant activity. J. Microbiol. Methods.

[18] Swatsitang P., Tucker G., Robards K., Jardine D. (2000). Isolation and identification of phenolic compounds in *Citrus sinensis*. Anal. Chim. Acta.

[19] Ramachandran S., Fontanille P., Pandey A., Larroche C. (2008). Fed-batch production of gluconic acid by terpene-treated *Aspergillus niger* spores. Appl. Biochem. Biotechnol..

[20] Amin M.A., Abd El Rehim S.S., El-Lithy A.S. (2010). Pitting and pitting control of Al in gluconic acid solutions—Polarization, chronoamperometry and morphological studies. Corros. Sci..

[21] Duckstein S.M., Stintzing F.C. (2014). Comprehensive study of the phenolics and saponins from *Helleborus niger* L. leaves and stems by liquid chromatography/tandem mass spectrometry. Chem. Biodivers..

[22] Li Y., Warren J.T., Boysen G., Gilbert L.I., Gold A., Sangaiah R., Ball L.M., Swenberg J.A. (2006). Profiling of ecdysteroids in complex biological samples using liquid chromatography/ion trap mass spectrometry. Rapid Commun. Mass Spectrom..

[23] Stevens J.F., Reed R.L., Morré J.T. (2008). Characterization of phytoecdysteroid glycosides in meadowfoam (*Limnanthes alba*) seed meal by positive and negative ion LC-MS/MS. J. Agric. Food Chem..

[24] Wang Y.H., Avula B., Jadhav A.N., Smillie T.J., Khan I.A. (2008). Structural characterization and identification of ecdysteroids from *Sida rhombifolia* L. in positive electrospray ionization by tandem mass spectrometry. Rapid Commun. Mass Spectrom..

[25] Wainwright G., Prescott M.C., Lomas L.O., Webster S.G., Rees H.H. (1997). Development of a new high-performance liquid chromatography-mass spectrometric method for the analysis of ecdysteroids in biological extracts. Arch. Insect Biochem..

[26] Destrez B., Pinel G., Bichon E., Monteau F., Lafont R., Le Bizec B. (2008). Detection of 20-hydroxyecdysone in calf urine by comparative liquid chromatography/high-resolution mass spectrometry and liquid chromatography/tandem mass spectrometry measurements: Application to the control of the potential misuse of ecdysteroids in cattle. Rapid Commun. Mass Spctrom..

[27] Miyashita M., Matsushita K., Nakamura S., Akahane S., Nakagawa Y., Miyagawa H. (2011). LC/MS/MS identification of 20-hydroxyecdysone in a scorpion (*Liocheles australasiae*) and its binding affinity to *in vitro*-translated molting hormone receptors. Insect Biochem. Mol..

[28] Laghari A.Q., Memon S., Nelofar A., Laghari A.H. (2013). *Tecomella undulate* G. Don: A rich source of flavonoids. Ind. Crop. Prod..

[29] Atoui A.K., Mansouri A., Boskou G., Kefalas P. (2005). Tea and herbal infusions: Their antioxidant activity and phenolic profile. Food Chem..

[30] Zhao Y., Chen P., Lin L., Harnly J.M., Yu L.L., Li Z. (2011). Tentative identification, quantitation, and principal component analysis of green pu-erh, green, and white teas using UPLC/DAD/MS. Food Chem..

[31] Kajdžanoska M., Gjamovski V., Stefova M. (2010). HPLC-DAD-ESI-MS^n^ identification of phenolic compounds in cultivated strawberries from Macedonia. Maced. J. Chem. Chem. Eng..

[32] Karioti A., Bilia A.R., Skaltsa H. (2010). *Quercus ilex* L.: A rich source of polyacylated flavonoid glucosides. Food Chem..

[33] Seeram P.N., Lee R., Scheuller S.H., Heber D. (2006). Identification of phenolic compounds in strawberries by liquid chromatography electrospray ionization mass spectroscopy. Food Chem..

[34] Mellou F., Lazari D., Skaltsa H., Tselepis A.D., Kolisis F.N., Stamatis H. (2005). Biocatalytic preparation of acylated derivatives of flavonoid glycosides enhances their antioxidant and antimicrobial activity. J. Biotechnol..

[35] Van der Doelen G.A., van den Berg K.J., Boon J.J., Shibayama N., Renè De La Rie E., Genuit W.J.L. (1998). Analysis of fresh triterpenoid resins and aged triterpenoid varnishes by high-performance liquid chromatography–atmospheric pressure chemical ionisation (tandem) mass spectrometry. J. Chromatogr. A.

[36] Chen Q., Zhang Y., Zhang W., Chen Z. (2011). Identification and quantification of oleanolic acid and ursolic acid in Chinese herbs by liquid chromatography—Ion trap mass spectrometry. Biomed. Chromatogr..

[37] Huang L., Chen T., Ye Z., Chen G. (2007). Use of liquid chromatography–atmospheric pressure chemical ionization-ion trap mass spectrometry for identification of oleanolic acid and ursolic acid in *Anoectochilus roxburghii* (wall.) Lindl. J. Mass Spectrom..

[38] Li Y.J., Wei H.L., Qi L.W., Chen J., Ren M.T., Li P. (2010). Characterization and identification of saponins in *Achyranthes bidentata* by rapid-resolution liquid chromatography with electrospray ionization quadrupole time-of-flight tandem mass spectrometry. Rapid Commun. Mass Spectrom..

[39] Liu X.R., Zheng X.F., Ji S.Z., Lv Y.H., Zheng D.Y., Xia Z.F., Zhang W.D. (2010). Metabolomic analysis of thermally injured and/or septic rats. Burns.

[40] Yang H., Lin W., Zhang J., Lin W., Xu P., Li J., Ling X. (2014). Metabonomic analysis of the toxic effects of TM208 in rat urine by HPLC-ESI-IT-TOF/MS. J. Chromatogr. B.

[41] Deng J., Yang Y. (2013). Chemical fingerprint analysis for quality assessment and control of Bansha herbal tea using paper spray mass spectrometry. Anal. Chim. Acta.

[42] Li J., Qi H., Qi L.W., Yi L., Li P. (2007). Simultaneous determination of main phytoecdysones and triterpenoids in radix achyranthis bidentatae by high-performance liquid chromatography with diode array-evaporative light scattering detectors and mass spectrometry. Anal. Chim. Acta.

[43] Destrez B., Pinel G., Monteau F., Lafont R., Le Bizec B. (2009). Detection and identification of 20-hydroxyecdysone metabolites in calf urine by liquid chromatography-high resolution or tandem mass spectrometry measurements and establishment of their kinetics of elimination after 20-hydroxyecdysone administration. Anal. Chim. Acta.

[44] Sánchez-Rabaneda F., Jáuregui O., Casals I., Andrés-Lacueva C., Izquierdo-Pulido M., Lamuela-Raventós R.M. (2003). Liquid chromatographic/electrospray ionization tandem mass spectrometric study of the phenolic composition of cocoa (*Theobroma cacao*). J. Mass Spectrom..

[45] Singh A.P., Wilson T., Luthria D., Freeman M.R., Scott R.M., Bilenker D., Shah S., Somasundaram S., Vorsa N. (2011). LC-MS–MS characterisation of curry leaf flavonols and antioxidant activity. Food Chem..

[46] Hvattum E., Ekeberg D. (2003). Study of the collision-induced radical cleavage of flavonoid glycosides using negative electrospray ionization tandem quadrupole mass spectrometry. J. Mass Spectrom..

[47] Gouveia S., Castilho P.C. (2011). Characterisation of phenolic acid derivatives and flavonoids from different morphological parts of *Helichrysum obconicum* by a RP-HPLC–DAD-(−)–ESI-MS*^n^* method. Food Chem..

[48] Castillo-Muñoz N., Gómez-Alonso S., García-Romero E., Gómez M.V., Velders A.H., Hermosín-Gutiérrez I. (2009). Flavonol 3-*O*-glycosides series of *Vitis vinifera* cv. Petit Verdot red wine grapes. J. Agric. Food Chem..

[49] Abu-Reidah I.M., Arráez-Román D., Quirantes-Piné R., Fernández-Arroyo S., Segura-Carretero A., Fernández-Gutiérrez A. (2002). HPLC–ESI-Q-TOF-MS for a comprehensive characterization of bioactive phenolic compounds in cucumber whole fruit extract. Food Res. Int..

[50] Falcão S.I., Vale N., Gomes P., Domingues M.R.M., Freire C., Cardoso S.M., Vilas-Boas M. (2012). Phenolic profiling of Portuguese propolis by LC–MS spectrometry: Uncommon propolis rich in flavonoid glycosides. Phytochem. Anal..

[51] Zehl M., Braunberger C., Conrad J., Crnogorac M., Krasteva S., Vogler B., Beifuss U., Krenn L. (2011). Identification and quantification of flavonoids and ellagic acid derivatives in therapeutically important Drosera species by LC–DAD, LC–NMR, NMR, and LC–MS. Anal. Bioanal. Chem..

[52] Ferreres F., Gil-Izquierdo A., Vinholes J., Silva S.T., Valentão P., Andrade P.B. (2012). *Bauhinia forficata* link authenticity using flavonoids profile: Relation with their biological properties. Food Chem..

[53] Ramirez J.E., Zambrano R., Sepúlveda B., Simirgiotis M.J. (2014). Antioxidant properties and hyphenated HPLC-PDA-MS profiling of chilean *Pica* mango fruits (*Mangifera indica* L. Cv. piqueño). Molecules.

[54] Gouveia S.C., Castilho P.C. (2009). Analysis of phenolic compounds from different morphological parts of *Helichrysum devium* by liquid chromatography with on-line UV and electrospray ionization mass spectrometric detection. Rapid Commun. Mass Spetrom..

[55] Plazonić A., Bucar F., Maleš Ž., Mornar A., Nigović B., Kujundžić N. (2009). Identification and quantification of flavonoids and phenolic acids in burr parsley (*Caucalis platycarpos* L.), using high-performance liquid chromatography with diode array detection and electrospray ionization mass spectrometry. Molecules.

[56] Kite G.C., Veitch N.C. (2011). Identification of common glycosyl groups of flavonoid *O*-glycosides by serial mass spectrometry of sodiated species. Rapid Commun. Mass Spectrom..

[57] Yang W.Z., Ye M., Qiao X., Liu C.F., Miao W.J., Bo T., Tao H.Y., Guo D.A. (2012). A strategy for efficient discovery of new natural compounds by integrating orthogonal column chromatography and liquid chromatography/mass spectrometry analysis: Its application in *Panax ginseng*, *Panax quinquefolium* and *Panax notoginseng* to characterize 437 potential new ginsenosides. Anal. Chim. Acta.

[58] Sun X., Chen P., Cook S.L., Jackson G.P., Harnly J.M., Harrington P.B. (2012). Classification of cultivation locations of *Panax quinquefolius* L samples using high performance liquid chromatography–electrospray ionization mass spectrometry and chemometric analysis. Anal. Chem..

[59] Yan Z., Lin G., Ye Y., Wang Y., Yan R. (2014). Triterpenoid saponins profiling by adducts-targeted neutral loss triggered enhanced resolution and product ion scanning using triple quadrupole linear ion trap mass spectrometry. Anal. Chim. Acta.

[60] Sha Y., Yan M.C., Liu J., Liu Y., Cheng M.S. (2008). Facile synthesis of oleanolic acid monoglycosides and diglycosides. Molecules.

[61] Waffo-Téguo P., Voutquenne L., Thoison O., Dumontet V., Nguyen V.H., Lavaud C. (2004). Acetylated glucuronide triterpene bidesmosidic saponins from *Symplocos glomerata*. Phytochemistry.

